# A big night out getting bigger: Alcohol consumption, arrests and crowd numbers, before and after legislative change

**DOI:** 10.1371/journal.pone.0218161

**Published:** 2019-06-20

**Authors:** Grant J. Devilly, Leanne Hides, David J. Kavanagh

**Affiliations:** 1 School of Applied Psychology, Griffith University, Queensland, Australia; 2 Griffith Criminology Institute, Griffith University, Queensland, Australia; 3 Centre for Youth Substance Abuse Research (CYSAR), Lives Lived Well Group, School of Psychology, University of Queensland, Queensland, Australia; 4 School of Psychology and Counselling, Queensland University of Technology, Queensland, Australia; University Hospital Carl Gustav Carus, Technische Universitat Dresden, GERMANY

## Abstract

**Background:**

Restrictive practices on alcohol sales in entertainment districts have been introduced to reduce alcohol-related violence in youth. On 1^st^ July 2016, the Queensland State Government (Australia) imposed a 2-hour reduction in trading hours for alcohol sales in venues within specific night-time entertainment districts (NEDS; from 5am to 3am), a reduction in maximum trading hours for venues outside NEDs (with a maximum 2am closing time), the banning of ‘rapid intoxication drinks’ (e.g. shots) after 12am, and no new approvals for trading hours beyond 10pm for the sale of takeaway alcohol. No independent study has evaluated general levels of intoxication, crowd numbers, fear of violence, and illicit substance use as people enter and exit NEDS, both before and after the introduction of restrictive legislation. Further, no study has assessed the impact using matched times of the year in a controlled study and also assessed actual assault rates as recorded by the police.

**Method:**

We conducted 3 studies–randomly breath-testing patrons for alcohol, as they entered and exited NEDs. Study 1 assessed patrons’ (n = 807) breath approximated blood alcohol concentration (BrAC) and predictions of how the legislation would change their drinking habits before the legislation was enacted. Study 2 assessed crime statistics and patrons’ BrAC levels and drug taking reports on an equivalent night, one year apart–before (n = 497) and after (n = 406) the new legislation. Study 3 was a test of the generalisation of Study 2 with two months of survey and BrAC data collected as people entered and exited the NEDs over two consecutive years before (n = 652 and n = 155) and one year after (n = 460) the new legislation. In Study 3 we also collected crime statistics and data on people leaving the entertainment district one year before (n = 502) and one year after (n = 514) the legislative change.

**Findings:**

People predicted that the legislation would lead to them drinking more alcohol before they entered town or make little change to their drinking habits. Baseline data over the 2 years before the legislation (Study 3) demonstrated stable preloading rates and BrAC at entry to the NEDs. However, after the introduction of the legislation patrons entered the NEDs systematically later and increased their alcohol preloading. People were substantially more inebriated as they entered the NEDs after the legislative change, with approximately 50% fewer people not preloading after the new laws. Exit BrAC was less consistent but showed some evidence of an increase. Crime statistics and patrons’ self-reported experiences of violence did not change.

**Interpretation:**

Legislation that does not specifically adapt to the cultural shift of preloading and take local conditions into account will be unsuccessful in reducing alcohol consumption. Such legislation is unlikely to meaningfully change assault rates in youth.

## Introduction

Binge drinking of alcohol (imbibing 5 or more drinks in one sitting [1]) is a global phenomenon that increases health risks in those who take part, whether that be in North America [2], the United Kingdom [3], Africa [4] or Asian and Caribbean countries [5]. These health risks include cancer, epilepsy, respiratory problems, liver failure and poor mental health. Binge drinking also disproportionately affects the developing brain of adolescents [6], and a disproportionate number of young people attend night-time entertainment districts (NEDs) where they engage in binge drinking, both before going out [7] and whilst out [3]. While a recent focus of research has quite rightly been aimed at discovering more about the trajectories that lead to alcohol abuse in adolescents [8]^,^ there has been a call for research which is more representative of low SES adolescents [9]. The current research focuses on a representative sample of youth in Australian NEDs, but has currency in all countries where youth congregate in similar venues.

In addition to medical health risks, ‘Alcohol fuelled violence’ is an oft-cited reason for temperance legislation and efforts to restrict opening hours of establishments selling alcohol. In Australia, this approach to violence reduction in NEDS has led to significant scientific [10, 11] and political [12] controversy. It is undisputed that there are more assaults and violent acts in Australian NEDs that stay open late and where alcohol is available, than in districts where alcohol is not available or where the clubs have earlier closing hours [13]. However, we are lacking data on whether assaults decrease as a result of reduced alcohol availability, or whether violence reduction is attributable to a decreased density of patrons in NEDs. We are also lacking convincing evidence that reductions in opening hours lead to a decrease in the general level of inebriation. Recent legislative changes in Queensland, Australia, provided a crucible in which we could test the effects of reduced drinking hours on inebriation and violence in NEDs.

In late 2015, the Queensland Government announced plans to introduce a “Tackling Alcohol-Fuelled Violence Amendment Bill”. Before this legislation, clubs were able to sell alcohol until 5am, with a 3am lockout. This lockout meant that after 3am people could not enter clubs, but those already inside could stay until 5am. The new legislation imposed a 2-hour reduction in trading hours for alcohol sales in venues within specific NEDs (from 5am to 3am), a reduction in maximum trading hours for venues outside NEDs (with a maximum 2am closing time), the banning of ‘rapid intoxication drinks’ (e.g. shots) after 12am, and no new approvals for trading hours beyond 10pm for the sale of takeaway alcohol. These aspects of the legislation came into effect on 1^st^ July 2016. This change in policy allowed an independent research team with longitudinal data to test new hypotheses and clarify the results of older studies.

In their seminal study, Kypri, Jones, McElduff, & Barker (2011) [14] explored the impact of introducing an earlier (3am) closing time and a (1.30am) lockout in the central business district (CBD) of Newcastle (Australia) on numbers of assaults, compared with a control setting in a nearby location (Hamilton). While they found a decrease in the number of assaults in Newcastle compared with Hamilton following the introduction of reduced licensing hours, this could have been related to reductions in the number of patrons in the area, due to the lockout, and alcohol-related assaults being displaced to other places or settings. Any link between reductions in the number of assaults and the average level of inebriation in the NED is unclear, as it was not assessed. The current research programme built upon this previous research by assessing average inebriation levels and street numbers both before and after legislative changes that reduced alcohol availability in the NEDs of a large Australian city. The impact of these legislative changes on perceptions of violence in the NEDs and their attitude towards entering these districts were also assessed.

Devilly, Allen & Brown (2017) [7] recently reported on alcohol preloading behaviours in NEDs in Queensland, Australia. Preloading is the act of consuming alcohol or drugs before entering the NEDs and has become a growing phenomenon in westernised countries, particularly in the UK and USA [15]. Devilly et al found that 79% of respondents had preloaded with alcohol, and that 71% returned a Breath Alcohol Concentration (BrAC) greater than zero. Those who preloaded with alcohol before entry into the NEDs had an average BrAC of .071% pure ethanol (n = 1,952; .073% for males and .068% for females). The major reason given for preloading was ‘to socialise’, followed by ‘to save money’. Preloading behaviours were associated with increased reports of harm to oneself and others, either through the preloading itself, or through increased violence or riskier sexual practices. Preloading levels were only marginally related to the time of night that patrons entered the NEDs (*r* = .12) and the location of preloading (private residences or suburban bars) had no significant impact on inebriation levels. There is also data that patrons’ BrAC at the end of the night is highly correlated to their entry BrAC, even when taking gender, personality and demographics (i.e., age and body mass index) into account [16]. If the trend towards preloading were to intensify, and there were no incentives for patrons to come into the NED earlier to counteract this preloading, then the average intoxication level of the city may actually rise. Given the presence of a growing preloading trend [15], and that people’s motivations to drink satisfies inherent urges [17], we speculated that reducing the availability of alcohol later in the night may increase the perceived intrinsic value of alcohol and make it more desirable. Evidence for this cognitive bias (the ‘scarcity heuristic’) is found in Lynn’s (1989) [18] treatise, in which participants assumed a bottle of wine was more expensive when it was scarce. Scarcity increased both the perceived expensiveness and desirability of the wine, but only when the real cost of the wine was unknown. People were also more willing to buy the wine if they did not know how much it cost, or when it was a gift for a friend.

This ‘scarcity heuristic’ may arise from a restriction approach to alcohol in NEDs via two potentially countervailing effects: alcohol as an issue is brought into people’s consciousness and may be valued higher, which would prime people to increase their drinking; but perhaps also more expensive, which may decrease drinking. A likely solution would be for people to begin or increase their alcohol intake while it is cheap (i.e., before entering the NED) and subsequently moderate their drinking when it is expensive (i.e., once in the NED, and irrespective of whether they preloaded or not). This could occur under a context of determined drunkenness, where youth predominantly abstain from alcohol during the beginning of the week, but binge in the late week / weekend [19]. In fact, it may be that the scarcity heuristic even creates this ‘determined drunkenness’ in some new people. It could also be possible that ‘unit time’ to drink becomes what is more scarce and, in a preloading culture that punishes early entry to the NEDs with little to no socialisation, people start drinking or drink more during this preloading period. Indeed, there may be other models to guide expectations (e.g., the Availability Theory, particularly legal availability, as explained by Wagenaar and Perry, 1994 [20]), but we find the scarcity heuristic to be an explanation that is parsimonious with legislation that legally restricts alcohol sales in a preloading culture.

Following this heuristic, we would expect preloading to increase, raising the intoxication level of patrons entering NEDs. This would be particularly concerning, given the high unique variance (between 30% and 40% dependent upon gender) in the relationship between intoxication levels at NED entry and exit [16]. We would, therefore, expect that restricted trading hours may have the unintended effects of patrons being more inebriated at the beginning of their night out, and that at the end of the night they would either have the same or higher blood alcohol readings than before the legislation. This would particularly be the case if people titrate their drinking to reach a certain level of inebriation before stopping or slowing down. In consequence, we did not expect the changes in legislation to have their intended effects of reducing the number of assaults and the public’s fear of assault due to inebriation.

Since we had collected data before the legislation was proposed and continued collecting it after the legislation came into effect, we also had an opportunity to gauge people’s drinking intentions relating to the legislation, and see whether these intentions were reflected in behavioural changes after the legislation was implemented. We expected respondents to say that the legislative changes would not lead them to come to venues earlier (despite fewer hours being available for drinking), but that they instead would say that they would drink more before coming out and drink at a greater rate whilst out [18, 16].

### Research method overview

All procedures were cleared by the Griffith University Human Research Ethics Committee (ref: PSY/71/14/HREC & 2015/704). Pooled data from people participating in 3 related projects with a similar methodology was used in this paper. These projects were the ‘SmartStart’, ‘Last Drinks’ and ‘What’s On Board’ projects. All participants gave verbal assent to take part in the research, which was gathered anonymously. Each participant was given an identification number and research card so that they could view the research website at a later time and request that their data be withdrawn from the study (for example, when they had become sober).

The ‘SmartStart’ project looked predominantly at preloading and risk-taking behaviours before entry into the NEDs (see Devilly, Allen & Brown, 2017 [7], for more details). This ran from 21^st^ August 2014 to 27^th^ February 2015, assessing 3,200 participants across major NEDs in South East Queensland from Thursday nights to Sunday mornings, and was conducted with a police presence to give credibility to the research team and to provide security for the researchers (although all data were collected anonymously and away from the police gaze). During testing we administered both a long and short version of a questionnaire. The short version enabled rapid data collection on key questions from multiple friends of the person completing the longer questionnaire.

It became evident that the security aspect of the police presence was unnecessary, and our later projects did not have a police presence during data collection from patrons. Recent research in the same context has demonstrated that a police presence does not affect the sampling of patrons in any specific direction, with similar inebriation rates being recorded in the two circumstances [11].

The ‘Last Drinks’ project looked at both ends of the night, assessing people’s inebriation level as they entered and exited the NEDs, again from Thursday evening to Sunday morning [16]. This ran from 31^st^ October 2015 to 26^th^ June 2016, and assessed 1,110 participants at entry and 3,017 participants as they exited NEDs. Other specialised studies targeting specific events (e.g., the University ‘Toga Party’ and Royal Exhibition Horse Races) were also run through our Last Drinks project but these data are not reported in the current paper. We also ran the 2016 arm of our study (called the ‘Big Night Out’) under this project, which is detailed further below under study 2.

Our third project, ‘What’s On-Board’, was predominantly aimed at assessing the drug culture in South East Queensland. We collected data on Thursday, Friday, and Saturday nights from 16^th^ September 2016 and data collection is ongoing. In that study we also collected alcohol data at NED entry and exit. As of the 7^th^ July 2017, we had assessed 988 participants at entry and 787 at exit. We also ran the 2017 arm of the ‘Big Night Out’ study under that project.

Data on estimated numbers at venues for both the 2016 and 2017 arms of Big Night Out were obtained by a police Inspector, who visited clubs throughout the night and asked for numbers of people inside the establishments. He did not interact with participants.

In all our studies we used breathalysers demonstrated to be reliable and valid with this population (platinum fuel-celled Alcolizer LE5s [21]) and QuickTapSurvey iPad questionnaires [22]. University researchers wore black Griffith University Polo shirts. In all studies, we approached every fourth person (or group) and asked them whether they would like a breathalyser test and do a quick survey about their drinking that night, as part of a research study. If they refused, then this was recorded as a refusal and the next person who passed was asked to take part. When a patron did not want to complete a questionnaire but wished to be breathalysed, they were offered the breathalysing as a community service, free of any expectation to complete a questionnaire. The participants held an iPad to complete the survey unless they were too drunk to do so without assistance. In the rare cases in which this occurred, the researcher held the iPad and read the questions aloud. At the end of the questionnaire the researcher breathalysed the participant, after ensuring that their last drink was at least 5 minutes prior to testing.

All breathalysers were calibrated to a 2100:1 partition ratio [23]. The breathalysers measure breath alcohol concentration (measured as grams of ethanol per 210 litres of breath), and pass that through an algorithm (multiplying by the partition ratio) to obtain a breath approximated blood alcohol concentration level (BrAC; measured as grams of ethanol per 100ml of arterial blood). We used the Alcolizer LE5, an instrument that uses an electro-chemical fuel-cell (platinum) to detect quantities between .000 - .500 BrAC with an accuracy of at least ±.01 at .100 BrAC g/100ml (generated from a breath sample). As a frame of reference, the legal drink driving limit in Australia is < .05 BAC g/100ml. The LE5 is certified by Australian standard 3547 and used by law enforcement agencies throughout Australia and South East Asia.

For studies 2 and 3 we also collected arrest rates from the Queensland Police Service Crime Map (https://www.police.qld.gov.au/forms/crimestatsdesktop.asp). This online resource allows any member of the public to examine levels of specific crime types within a postcode or area. We selected good order offences (anything that potentially disrupts the peace of a public place, including public nuisance, obstruction of police and public urination) and assaults as our major indicators of crime in the NEDs potentially related to alcohol. This resource does not allow for a breakdown of crime rates by time of day but goes from 12.00am until 11.59pm of the day in interest. “From” and “To” dates allows derivation of crime statistics over a period of time (e.g., January 1^st^ to February 28^th^ in 2016 and 2017).

## Study 1

### Aim

Study 1 examined NED patrons’ attitudes toward the introduction of the 2016 legislation and its impact on BrAC levels.

### Method and participants

Study 1 participants were drawn from our larger Last Drinks project. A subsample (n = 368) completed a questionnaire on entry to NEDs, which included three questions about the upcoming change in legislation to a 3am cessation of alcohol sales in the NEDS (See Table 1). Another 443 participants completed a shorter questionnaire at entry to the NED which asked one question about their opinions concerning the new legislation (Question 1, Table 1), providing a total sample of 811 for that question.

**Table 1:** Patron reports of how they expected the legislative changes to affect their drinking behaviour.

Question	Answers	Male N (%)	Female N (%)	All N (%)	BrAC $\bar{x}$ (sd, N) / % zeros^a^^,^^b^
1. It seems likely that the laws may change so that entertainment venues will close at 3 AM and stop selling alcohol before this time. Will this affect what time you come into the entertainment district? Gender *χ*^2^(*N* = 811, *df* = 2) = 6.9, *p* = .03, *Phi* = 0.092	Yes: arrive earlier	180 (48.13%)	191 (43.71%)	371 (45.75%)	.053 (.049, 370) / 45.19%
	No	171 (45.72%)	232 (53.09%)	403 (49.69%)	.052 (.05, 401) / 50.96%
	Yes: arrive later	23 (6.15%)	14 (3.20%)	37 (4.56%)	.066 (.061, 36) / 3.85%
2. Will the change in laws affect how much alcohol you drink before coming out? Gender *χ*^2^(*N* = 368, *df* = 2) = 2.75, *p* = .25, *Phi* = 0.086	Yes: Drink less before coming out	5 (2.98%)	2 (1%)	7 (1.90%)	.042 (.052, 6) / 3.33%
	No	86 (51.19%)	114 (57%)	200 (54.35%)	.052 (.048, 199) / 57.78%
	Yes: Drink more before coming out	77 (45.83%)	84 (42%)	161 (43.75%)	.053 (.047, 159) / 38.89%
3. Will the change in laws affect how much you drink whilst you are out? Gender *χ*^2^(*N* = 368, *df* = 2) = 3.30, *p* = .19, *Phi* = 0.095	Yes: Drink less whilst out	21 (12.50%)	16 (8%)	37 (10.05%)	.044 (.044, 35) / 11.11%
	No	92 (54.76%)	126 (63%)	218 (59.24%)	.052 (.048, 216) / 64.44%
	Yes: Drink more whilst out	55 (32.74%)	58 (29%)	113 (30.71%)	.054 (.047, 113) / 24.44%

Note

^a^ = Percentage of patrons who blew zero on BrAC that answered that question in that way (e.g., on question 1. 45.19% of the 208 people who blew a BrAC of zero said “yes: arrive earlier”)

^b^ = 208 of the 807 participants (26%) answering Question 1 had a BrAC of zero, as did 90 of the 364 (25%) answering Questions 2 and 3.

### Results

We found very few differences between the responses of males and females. Males were less likely than females to report that the changes in legislation would change their behaviour, particularly in relation to NED entry time, although the effect size was very small (*χ*^2^(*N* = 811, *df* = 2) = 6.9, *p* = .03, *Phi* = .092). No gender differences on intentions to drink more before or whilst in the NED were found. There was almost an even split between participants reporting that they would come into the NEDs earlier following the introduction of the new legislation and reporting that it would make no difference to their behaviour. Respondents also said that the legislative changes would either lead to them preloading more or would make no difference to their pre-drinking. While over half predicted that the changes would make no difference to their drinking whilst out (59%), more than 30% thought that they would drink more (Table 1).

Associations between BrAC readings and attitudes toward the new legislation were also examined. People with higher BrACs tended to predict that they would arrive later, drink more before coming out and drink more whilst out, although it should be noted that these did not reach the .05 level of significance. People with a BrAC of zero were most likely to say that the legislative changes would not affect their drinking behaviour, followed by stating that they would arrive into town earlier, drink more before they come out and drink more whilst out. In effect, people with a BrAC of zero were more likely to see the legislative changes as not impacting their drinking behaviour either before going out or whilst out (*p* < .05).

## Study 2

### Aim

Study 2 reports on the impact of the legislation on patrons’ preloading, BrAC readings at NED entry and exit, assaults, street and venue numbers on one night, in the 12 months before and after the 2016 legislative changes.

### Method and participants

Study 2 participants were obtained from our Big Night Out study, which was a single-night snapshot across major areas in the city of Brisbane. This involved us breathalysing people on entry and exit to the same NED locations, on a Saturday night one year apart (before and after the 2016 legislative changes). We started data collection at 7pm on both occasions and completed our data collection when there were no more people answering the questionnaire in the NEDs for more than 20 minutes. The 2016 data were collected on 2^nd^ April (week 13 of the year) and the 2017 data were collected on 25^th^ March (week 12). These dates were in the same University semester week over the two years (weeks 4 to 5), had similar temperatures in Brisbane (2016 = 19.9^o^c– 30.4 ^o^c; 2017 = 21.5 ^o^c– 28 ^o^c), allowed for a full contingent of researchers to be available and were not biased by large sporting or other State events. The four locations for data acquisition were: Brisbane CBD and its Treasury Casino, the Caxton Street NED (1km west of the city centre), and the inner-city entertainment precinct of Fortitude Valley (1.6km north-east of the city centre). Street numbers were counted at exactly the same place and club numbers were obtained from the same nightclubs except one (Casablanca’s, which had closed by March 2017: for the 2017 data collection we, therefore, assessed numbers from the replacement club at the same location—Fritzenberger Brew Pub). Details on street and club numbers are provided below. Alcohol sales at the Treasury Casino in Brisbane come under the Casino Act and not the Liquor Licensing Act, and so were exempt from the 3am cessation of alcohol sales. To obtain as representative a sample as possible, we sampled customers outside the Casino in both 2016 and 2017, ensuring that we asked whether participants had already been drinking in town. Data from people who had already been drinking in one of the Brisbane NEDs were omitted.

We obtained 184 entries and 313 exits in 2016, and 139 entries and 267 exits in 2017. In 2016, the first data collection at entry was at 8:14pm and the last entry collection was at 4:48am. Excluding people arriving at the Casino meant our last entry record was at 00:51am. In 2017, our first data collection for entry was at 8:44pm and the last entry collection was at 4:13am. Excluding people arriving at the Casino meant the last entry record was at 00:26am.

In 2016 our first exit data was gathered at 8:34pm and last exit data obtained at 5:07am. Excluding people who were leaving the Casino led to our last exit data still being at 5:07am. In 2017 the first exit data was gathered at 8:52pm and the last exit data obtained at 4:58am. Excluding people who were leaving the Casino led to the last exit data being at 4:46am.

In the Big Night Out study we collected numbers of patrons in the streets using a method seen as the gold standard in a recent validation study [24]. The street counts were conducted by a researcher standing at a specific place (one that captured representative pedestrian traffic for that area) and fixating their gaze at a specific point on the opposite side of the street. Using a hand-held tally-counter, every person who crossed that line of vision, irrespective of direction, led to an increased tally. This was conducted for 5-minute intervals at each location and each location was visited as often as possible, as the researcher traced a 9km circular route around the city, escorted in an unmarked police car. The same researcher conducted the counts at the same locations in both 2016 and 2017. Thirteen street counts were conducted in both years. As there was already a 3am lockout before the new legislation came into effect, we felt it important to gauge not only the number of people in the street, but also the number of people in the clubs. Therefore, we also collected numbers of patrons in selected clubs at the various sites throughout the night. The club number estimates were obtained by a police officer who asked the door staff for the number of people in their clubs. In Australia, crowd estimates are required to prevent overcrowding and comply with fire safety legislation.

### Results

#### Surveys and breathalysers

During the Big Night Out we obtained a similar sample in 2016 and 2017 (Table 2). There was a similar ratio of genders (*χ*^2^(*N* = 323, *df* = 1) = .06, *p* = .42, *Phi* = .05), and the mean age of the participants was not significantly different (*t*(321) = -1.26, *p* = .21, *g* = -.14, *95%CI*:-.36,.083). In fact, ages were equivalent using a Two One-Sided t-Test [25] with 6 years set as meaningful (as per the Australian Bureau of Statistics National Health Survey categorisation for this age range; ABS, 2015 [26]; df = 301.68, t_upper_ -10.32 < -1.65, t_lower_ 7.79>1.65). Our refusal rates during the research were not significantly different (*χ*^2^(*N* = 1,072, *df* = 1) = 1.63, *p* = .20, *Phi* = .04). In 2016, we had 84 of 581 people (14.16%) refuse to take part when approached, and in 2017 we had 85 of 491 people (17.31%) refuse to take part.

**Table 2:** Big night out entry & exit data.

		**2016**			**2017**		
**Entry or Exit**	**Variable**	**Males (N = 91)**	**Females (N = 93)**	**All (N = 184)**	**Males (N = 75)**	**Females (N = 64)**	**All (N = 139)**
Entry	Age in years $\bar{x}$ (sd)	22.75 (6.42)	22.96 (5.53)	22.85 (5.97)	24.25 (6.56)	23.03 (4.71)	23.69 (5.80)
Entry	^a^Time of Night $\bar{x}$ (SD; Median)	22.49pm (1.22; 22.49pm)	22.29pm (1.05; 22.29pm)	22.39pm (1.15; 22.44pm)	22.31pm (1.18; 22.18pm)	22.13pm (0.86; 22.11pm)	22.23 (1.05; 22.15pm)
Entry	To nearest hour, when start to get ready? Median (Mode)	7pm (8pm)	7pm (6pm)	7pm (6pm)	7pm (8pm)	6pm (6pm)	6pm (6pm)
Entry	^b^Self-Reported Alcohol Preloading % (N)	80.22 (73)	88.17 (82)	84.24 (155)	90.67 (68)	92.19 (59)	91.37 (127)
Entry	^c^Number Of Standard Drinks? $\bar{x}$ (SD; Median)	5.90 (5.15; 5)	4.44 (3.34; 4)	5.16 (4.38; 4)	6.04 (3.61; 6)	5.03 (3.09; 5)	5.58 (3.41; 6)
Entry	^d^Number Of Standard Drinks if > 0? $\bar{x}$ (SD; Median; N)	7.36 (4.72; 6; 73)	5.04 (3.11; 4; 82)	6.13 (4.10; 5; 155)	6.66 (3.19; 6; 68)	5.46 (2.82; 5; 59)	6.10 (3.08; 6; 127)
Entry	Those admitting to having taken ‘party drugs’ before entry to NED. % (N)	7.69 (7)	4.30 (4)	5.98 (11)	8 (6)	1.56 (1)	5.04 (7)
Entry	^e^How affected by drink or drugs (1–5)? $\bar{x}$ (SD; Median)	2.14 (1.03; 2)	2.11 (0.82; 2)	2.13 (0.93; 2)	2.35 (0.89; 2)	2.28 (1.03; 2)	2.32 (0.96; 2)
Entry	People with a BrAC of 0%. % (N)	35.17 (32)	16.30 (15)	25.68 (47)	12.16 (9)	9.52 (6)	10.95 (15)
Entry	BrAC of People irrespective of preloading status. $\bar{x}$ (SD; Median; N)	.048 (.047; .044; 91)	.057 (.05; .052; 92)	.053 (.049; .046; 183)	.076 (.058; .072; 74)	.081 (.051; .081; 63)	.078 (.055; .075; 137)
Entry	BrAC Of Those With A BAC > 0% $\bar{x}$ (SD; Median; N)	.074 (.039; .068; 59)	.068 (.047; .059; 77)	.071 (.043; .061; 136)	.086 (.054; .092; 65)	.09 (.046; .093; 57)	.088 (.05; .093; 122)
		**2016**			**2017**		
		**Males (N = 182)**	**Females (N = 131)**	**All (N = 313)**	**Males (N = 160)**	**Females (N = 107)**	**All (N = 267)**
Exit	Age in years $\bar{x}$ (sd)	27.53 (7.78)	26.78 (8.11)	27.22 (7.91)	25.76 (8.61)	26.64 (9.82)	26.11 (9.11)
Exit	^a^Time of Night $\bar{x}$ (SD; Median)	2.28am (1.53; 2.51am)	2.16am (1.46; 2.17am)	2.23am (1.50; 2.50am)	2.39am (1.49; 3.11am)	2.13am (1.51; 2.18am)	2.29am (1.50; 2.58am)
Exit	To nearest hour when come into town? Median (Mode)	10pm (10pm)	10pm (10pm)	10pm (10pm)	10pm (11pm)	10pm (11pm)	10pm (11pm)
Exit	^c^Number Of Standard Drinks in town? $\bar{x}$ (SD; Median)	8.36 (5.47; 8)	6.6 (4.34; 6)	7.62 (5.09; 7)	9.55 (6.32; 9)	7.09 (3.94; 7)	8.56 (5.61; 8)
Exit	^d^Number Of Standard Drinks in town if > 0? $\bar{x}$ (SD; Median; N)	8.79 (5.25; 8; 173)	6.86 (4.22; 7; 126)	7.98 (4.93; 7; 299)	9.92 (6.15; 9; 154)	7.16 (3.9; 7; 106)	8.79 (5.51; 8; 260)
Exit	Those admitting to having taken ‘party drugs’ inside the NED. % (N)	7.14 (13)	8.40 (11)	7.67 (24)	17.5 (28)	6.54 (7)	13.11 (35)
Exit	^e^How affected by drink or drugs (1–5)? $\bar{x}$ (SD; Median; N)	2.48 (1.02; 2)	2.53 (1.09; 3)	2.50 (1.05; 3)	2.8 (1.03; 3)	2.65 (1.04; 3)	2.74 (1.03; 3)
Exit	People with a BrAC of 0%. % (N)	13.73 (25)	19.08 (25)	15.97 (50)	8.75 (14)	5.61 (6)	7.49 (20)
Exit	BrAC of People irrespective of alcohol use status. $\bar{x}$ (SD; Median; N)	.086 (.063; .081; 182)	.08 (.063; .078; 131)	.084 (.063; .081; 313)	.099 (.062; .096; 160)	.092 (.06; .084; 107)	.096 (.061; .093; 267)
Exit	BrAC Of Those With A BAC > 0% $\bar{x}$ (SD; Median; N)	.1 (.057; .094; 157)	.099 (.055; .095; 106)	.1 (.056; .094; 263)	.109 (.056; .102; 146)	.097 (.057; .089; 101)	.104 (.057; .096; 247)
Exit	^f^Fear of violence. $\bar{x}$; (SD Median; Mode)	1.18 (.52; 1; 1)	1.28 (.75; 1; 1)	1.22 (.62; 1; 1)	1.26 (.71; 1; 1)	1.19 (.53; 1; 1)	1.23 (.64; 1; 1)
	(1)—% (N)	86.81 (158)	85.5 (112)	86.26 (270)	83.75 (134)	86.92 (93)	85.02 (227)
	(2)—% (N)	10.44 (19)	6.11 (8)	8.63 (27)	11.25 (18)	8.41 (9)	10.11 (27)
	(3)—% (N)	1.1 (2)	3.82 (5)	2.24 (7)	0 (0)	3.74 (4)	1.5 (4)
	(4)—% (N)	1.65 (3)	4.58 (6)	2.88 (9)	5 (8)	.94 (1)	3.37 (9)

Note

^a^ = Mean, median and standard deviation in hours and minutes

^b^ = did you preload with alcohol before coming into the entertainment district tonight?

^c^ = including those who reported to have had no drinks before entering the NED

^d^ = for those stating they preloaded with alcohol only—set to a minimum of 1 and maximum of 30

^e^ = How affected do you feel by drink and / or drugs? (1) Not at all, (2) A Little, (3) Somewhat, (4) Very, (5) Extremely

^f^ = at any point tonight were you concerned that someone might hurt / attack you? (1) Not at all, (2) Maybe a little, in general, (3) Yes, I had a verbal argument, (4) Yes, I was physically touched against my will.

Given our prediction, based on our theoretical rationale and the results of study 1 (that people would preload more following the new legislation), a one-tailed test was used to assess preloading. A significant increase in self-reported preloading occurred from 2016 (84.24%) to 2017 (91.37%; *χ*^2^(*N* = 323, *df* = 1) = 3.63, *p* < .03, *Phi* = .11). Although the absolute value of the number of drinks self-reportedly consumed during preloading increased from 2016–2017, this effect was not statistically significant (p = .36).

**Fig 1:**
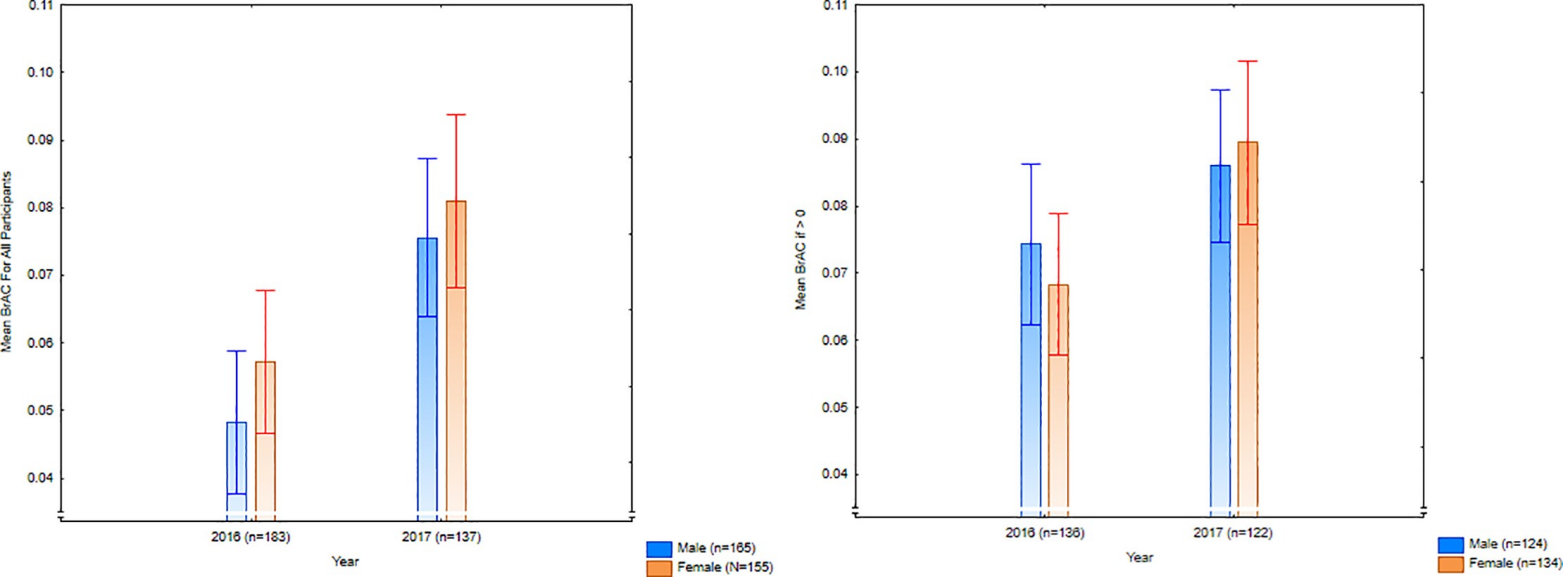
Entry mean breath alcohol concentration (with 95% confidence intervals) for the big night out, both before (2016) and after (2017) the legislative changes.

Fewer participants had a BrAC of zero at entry into the NEDs in 2017 (10.95%) compared with 2016 (25.68%; *χ*^2^(*N* = 323, *df* = 1) = 11.11, *p* = .0009, *Phi* = .19). Furthermore, irrespective of whether the participant had or had not preloaded, there was a significantly higher BrAC average after the legislation in 2017 compared with 2016, even when computing with a more conservative nonparametric statistic to account for the presence of zero readings in 2016 (Mann-Whitney U-Test *Z* = -4.36, *p* < .0001). Assuming the robustness of an ANOVA for non-normal data [27], using this approach further increased the statistical significance of this result and gave a medium effect size (*Hedges’ g =* .49, 95%CI:.27, .72). Data looking only at participants who had a BrAC reading above zero^cf 10^ demonstrated that drinkers were significantly more inebriated in 2017 due to preloading (*F*(1,256) = 8.23, *p* = .005, *Hedges’ g* = .36, 95%CI:.11, .6; Fig 1). Considering this increase in preloading, it is not surprising that patrons’ self-perception of being affected by alcohol or drugs also commensurately rose between 2016 and 2017 (*t*(321) = 1.79, *p*(1-tailed) = .04; *Hedges’ g* = .2). There was no difference in the number of people who admitted to taking party drugs across the years, with only small numbers reporting this behaviour.

At exit, we had similar samples in 2016 and 2017 based on age, gender, and the time of night they said they had arrived in town. Patrons in 2017 were significantly more likely to record a non-zero BrAC (*χ*^2^(*N* = 580, *df* = 1) = 9.77, *p* = .002, *Phi* = .13). While those who had a BrAC greater than 0 had similar average levels across the years, there was a general increase in BrAC at exit for the entire sample in 2017 (Mann-Whitney U-Test *Z* = -2.51, *p* = .01). The full exit sample also saw themselves as significantly more inebriated (*F*(1,578) = 7.04, *p* = .008), and were more likely to admit to taking party drugs inside the NEDs after the change in legislation (*χ*^2^(*N* = 580, *df* = 1) = 4.67, *p* = .03, *Phi* = .09). While the numbers admitting to the illegal behaviour of drug use were relatively small, this increase represented almost a doubling, with 24 out of 313 (7.67%) in 2016 versus 35 out of 267 (13.11%) in 2017. While the new legislation brought an earlier end to the night, the average time of night that we surveyed people leaving the NEDs was similar across years, suggesting that the patrons were, on average, not leaving the NEDs earlier (*t*(578) = -0.48, *p* = 0.63). However, closer inspection of these times shows that people did leave one of the NEDs (Caxton Street) significantly earlier in 2017 ($\bar{x}$ = 11.10pm, sd = 1 hour 11 minutes) compared with 2016 ($\bar{x}$ = 2am, sd = 1 hour 26 minutes), representing a very large effect size (*Hedges’ g* = 2.81).

#### Assaults

The new legislation was introduced to reduce alcohol related violence. Consequently, we were interested in patrons’ fear of assault during the night, and in the incidence of assaults and good order offences. As presented in Table 2, there was no meaningful change in experienced fear of assault during the night by our samples. Likewise, there was no significant difference over the specific days of data collection in reported assaults and good order offences between the two years, with numbers remaining low across time (Table 3).

**Table 3:** Big night out crimes against the person and good order offences.

		Good Order Offences	Assault
2016 = 2^nd^ April 16 2017 = 25^th^ March 17	CBD—2016	2	2
	CBD—2017	10	4
	Valley—2016	21	5
	Valley—2017	12	3
	Total—2016	23	7
	Total—2017	22	7

No sig diff for totals: (Yates’ Corrected *χ*^2^(*N* = 59, *df* = 1) = 0.06, *p* = .82, *Phi* = 0.01)

#### Street numbers

As a proportion of all participants observed from that year, there was no significant change from 2016 to 2017 in the number of people attending the NEDs between 9pm to 12 midnight (*χ*^2^(*N* = 2,912, *df* = 1) = 0.1, *p* = .38, *Phi* = .006), from 12am to 3am (*χ*^2^(*N* = 2,912, *df* = 1) = 2.26, *p* = .13, *Phi* = .03), or from 3am to 5am (*χ*^2^(*N* = 2,912, *df* = 1) = 2.22, *p* = .14, *Phi* = .03). However, the absolute number of people counted increased from 2016 to 2017 by 30.63% (see Table 4)

**Table 4:** Big night out street counts.

Location / Year	9pm– 12am Average N^o^ of People per 5 Mins (N^o^ of Counts)	12am– 3am Average N^o^ of People per 5 Mins (N^o^ of Counts)	3am– 5am Average N^o^ of People per 5 Mins (N^o^ of Counts)	Night Totals
Valley				
2016	349 (1)	443 (2)	178	**970**
2017	446.5 (2)	505 (1)	276.67 (3)	**1228.17**
CBD				
2016	37 (1)	34 (1)	20 (1)	**91**
2017	56 (1)	71 (1)	0 (1)^b^	**127**
Caxton Street				
2016	38.5 (2)	27 (1)	0 (1)^b^	**65.5**
2017	58	37 (1)	0 (1)^b^	**95**
Casino				
2016	103 (2)	- (0)^a^	33 (1)	**136**^**c**^
2017	138 (1)	13 (1)	61 (1)	**199**^**c**^
** Time Totals**				
** 2016**	**527.5**	**504**^**c**^	**231**	**1,262.5**^**c**^
** 2017**	**698.5**	**613**^**c**^	**337.67**	**1,649.17**^**c**^

Note

^a^ = Casino not visited during time frame

^b^ = Street empty

^c^ = Not including Casino 12–3 time frame numbers in the totals.

#### Club numbers

Table 5 presents the averaged numbers inside the clubs, as reported by the clubs to the police Inspector. Looking at the totals for the three timeframes from the same night clubs, it became evident that there were more people in the clubs after the legislation than before (*χ*^2^(*N* = 4,521, *df* = 2) = 135.32, two-tailed *p* < .0001, *Phi* = .17). We have not included the data from the Casino as the numbers given to the researchers in 2017 appeared extremely inaccurate. The researchers walked through the premises themselves to check the figures that had been given and estimated that the figures had been at least doubled by the security staff. We only decided to present the Casino data here at all because we wished to present a complete account of the research.

**Table 5:** Big night out club counts.

	Average Number of People Per Location Per Club (i.e., N/Total Counts)			
Location / Year	9–12 (N^o^ of Clubs / Total Counts)	12–3 (N^o^ of Clubs / Total Counts)	3–5 (N^o^ of Clubs / Total Counts)	Night Totals
Valley				
2016	210 (2 / 2)	667.5 (2 / 2)	220 (2 / 2)	**1097.5**
2017	292.33 (6 / 6), 295 (2 / 2)^a^	1220 (2 / 2)^a^	371.5 (6 / 6), 748 (2 / 2)^a^	**1883.83, 2263**^**a**^
CBD				
2016	185 (2 / 2)	220 (2 / 2)	35 (2 / 2)	**440**
2017	100 (2 / 2)	200.5 (2 / 2)	0 (2 / 2)^b^	**300.5**
Caxton Street				
2016	120 (2 / 4)	115 (2 / 2)	0 (2 / 2)^b^	**235**
2017	110 (2 / 2)	75 (2 / 2)	0 (2 / 2)^b^	**185**
Casino				
2016	3,000 (1 / 1)	2,100 (1 / 1)	700 (1 / 1)	**5,800**
2017	— (6,000 (1 / 1))^c^	— (2,500 (1 / 1))^c^	— (6,000 (1/1))^c^	
** Time Totals**				
** 2016**	**515**	**1,002.5**	**255**	**1772.5**
** 2017**	**502.33, 505**^**a**^	**1,495.5**	**371.5, 748**^**a**^	**2369.33, 2748.5**^**a**^

Note

^a^ = using only the same clubs as 2016

^b^ = Both clubs had shut for the night

^c^ = Casino data for 2017 unreliable and hence all totals and analyses do not include the Casino data.

### Discussion of studies 1 and 2

As expected in Study 1, people predicted that they would come into the NEDs earlier or keep to their current time regime after the introduction of the new laws. It should be noted that while there was already a 3am lockout in place when this data was collected, the clubs did not shut until 5am.

Further, participants in study 1 stated an intention to drink more before and during their time in the NEDs (or at least as much as before the legislative change). However, we found no association between BrAC readings and NED entry time, preloading or consumption whilst out. The results suggested that it was likely that preloading would increase following the legislative changes, with 43.75% of people predicting that they would preload more and only 1.90% predicting that they would preload less. A clarifying analysis of our SmartStart data [7] finds that people who preload tend to enter the NEDs significantly later than people who do not preload (*t*(2163) = 4.7, *p* < .001; Hedges’ *g* = 0.25). This would suggest that, in general, people would be likely to enter the NEDs later following the legislation, unless enticements to bring people out earlier were also introduced (e.g., allowing advertisements for ‘happy hour’). Further, in the absence of legislation to curtail excessive drinking towards the end of the night (e.g. compulsory displays of pricelists in drinking venues; cf. Lynn, 1989 [18]) it also seemed likely that, while in the NED, patrons would drink at least as much as before the legislation and possibly more.

In study 2, and consistent with people’s intentions in study 1 and our hypothesis from theory, people preloaded with more alcohol following the legislative changes. Further, as predicted a greater percentage of people appeared to be preloading (i.e., had a BrAC greater than zero on NED entry) following the legislative changes. Likewise, there was a smaller percentage of people who did not register a BrAC over zero when leaving the NEDs, and those who had been drinking appeared to have been drinking more than before the changes. This is consistent with the scarcity hypothesis [18].

Assault rates, good order offences and general levels of fear in participants did not appear to have been affected by the legislation. Anecdotally, the researchers did notice that people were emptying onto the streets (and some into taxi ranks) around the 3am alcohol sales cut-off. What violence did occur during the night seemed to be happening either as people entered the NED or around this 3am time point, but the police data did not allow this impression to be tested systematically. This hypothesis requires further research.

Street and club counts suggest little difference across legislation changes. If anything, it appeared that there were more people in the NEDs following the legislation and, when the cessation of alcohol occurred, they were all leaving the clubs and staying on the streets for some time. However, the smaller NED (Caxton Street) closed earlier and people congregated in a larger NED (Fortitude Valley).

During the street counts it came to the researchers’ attention that one of the nightclubs in the Valley was open for the last night before refurbishment, and a very large number of people were milling around the street count area due to the excessively long line-up to enter the club (which occurred from 11pm until 3am). We are not surprised, therefore, by the larger number of people who were counted in 2017 in the Valley across the night. However, even excluding the Valley location, one can see an increase in the Brisbane CBD. In contrast, the smaller NED of Caxton Street had virtually stopped functioning as an entertainment district after midnight. While the results point towards people not coming into the NEDs earlier and not leaving the NEDs when the clubs stopped selling alcohol at 3am, these results may have been influenced by a 3am ‘lockout’ (as opposed to a cessation of alcohol sales) that was in place in 2016 before the legislative changes. Therefore, it was necessary to also look at the number of people inside the clubs.

The number and time of street counts was somewhat dependent upon the flow of traffic and whether the police officer was called to intervene in any ongoing disorder, and the above results must, therefore, be interpreted with caution. Future studies that include street counts would benefit from having multiple personnel to conduct counts, to minimise variation due to extraneous factors, and enable calculation of the reliability of the observations.

Study 2 offered a snapshot of Brisbane both before and after legislative changes to curtail the sale of alcohol and reduce violence. However, it occurred on just one night, albeit in five simultaneous locations. It is possible that these results were aberrations, and it was necessary to check that the results were generalisable to other days.

## Study 3

### Aim

Study 3 reports on the impact of the legislation on patrons’ BrAC readings at NED entry and exit, and on assaults, from Thursday evening to Sunday morning over the same 2-month period, starting 18 months and 6 months before the 2016 legislative changes, and restarting 6 months after them.

### Method and participants

Study 3 participants were obtained over the months of January and February in the years 2015, 2016 and 2017. This allowed for baseline samples (2015 and 2016) to be compared with a sample collected post-legislation. All three studies had samples from Brisbane’s Fortitude Valley and CBD. We do not have refusal rates for all nights of our data collection, but previous research with our methodology has found a 14.67% refusal rate [5]. These data came from three separately funded research projects:

- 2015: From our SmartStart Project, there were 652 participants in January and February, of whom 347 were men and 305 women.
- 2016: From our ‘Last Drinks’ project, there were 155 participants in January and February, of whom 67 were men and 88 women.
- 2017: From our ‘What’s On-Board’ project, we recruited 460 participants in January and February, of whom 246 were men and 214 women.

### Results

#### Surveys and breathalysers

Although the samples before and after the legislation differed in average age (*F*(1,1263) = 7.27, *p* = .007), this represented only a small difference of 8.6 months (2015 & 2016 combined $\bar{x}$ = 22.03 years, sd = 4.7, N = 805; 2017 $\bar{x}$ = 21.31 years, sd = 4.41, N = 460; *Hedges’ g* = 0.16, 95%CI = 0.04, 0.27). Considering the legal age for buying alcohol in Australia is 18 years old, we do not see an 8-month difference at this age as significant. However, to check this, and assuming a non-trivial difference of 6 years (consistent with the Australian National Health Survey data range scales; ABS, 2015), this difference is easily within the range of equivalence (*df* = 1006.2, t_upper_ -19.65<-1.65, t_lower_ 25.01>1.65). In fact, it would require the non-trivial difference to be just 1.2 years for the data to display non-equivalence. The gender mix was comparable across the years (*χ*^2^(*N* = 1,267, *df* = 1) = .56, two-tailed *p* = .46, *Phi* = .02).

**Fig 2:**
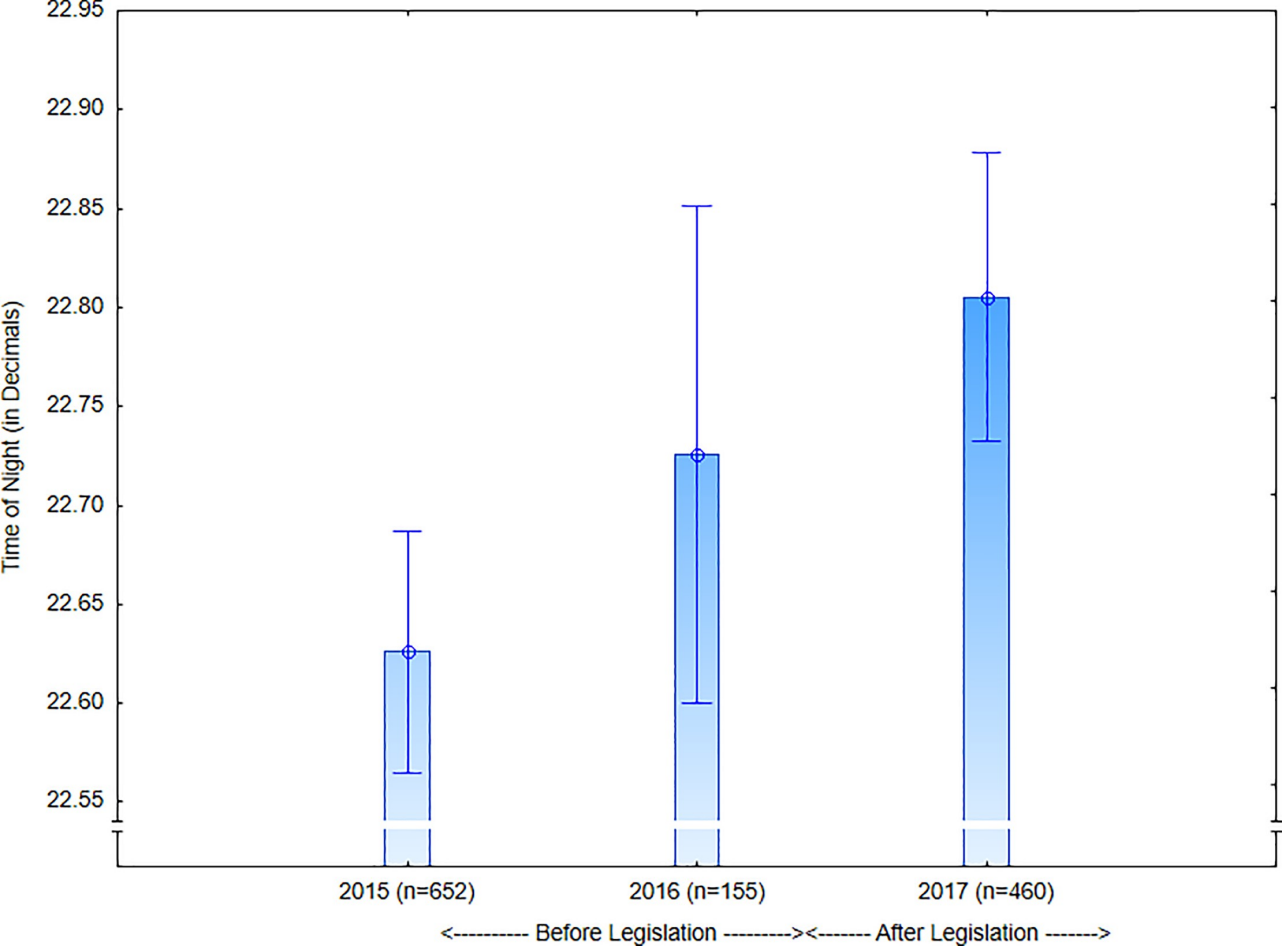
Time of night patrons entered the NEDs.

People entered the NEDs progressively later from 2015 to 2017, although the effect size was relatively small (*F*(2,1264) = 6.91, p = .001; *Hedges’ g* = 0.23, 95%CI: 0.11, 0.36; Fig 2).

**Fig 3:**
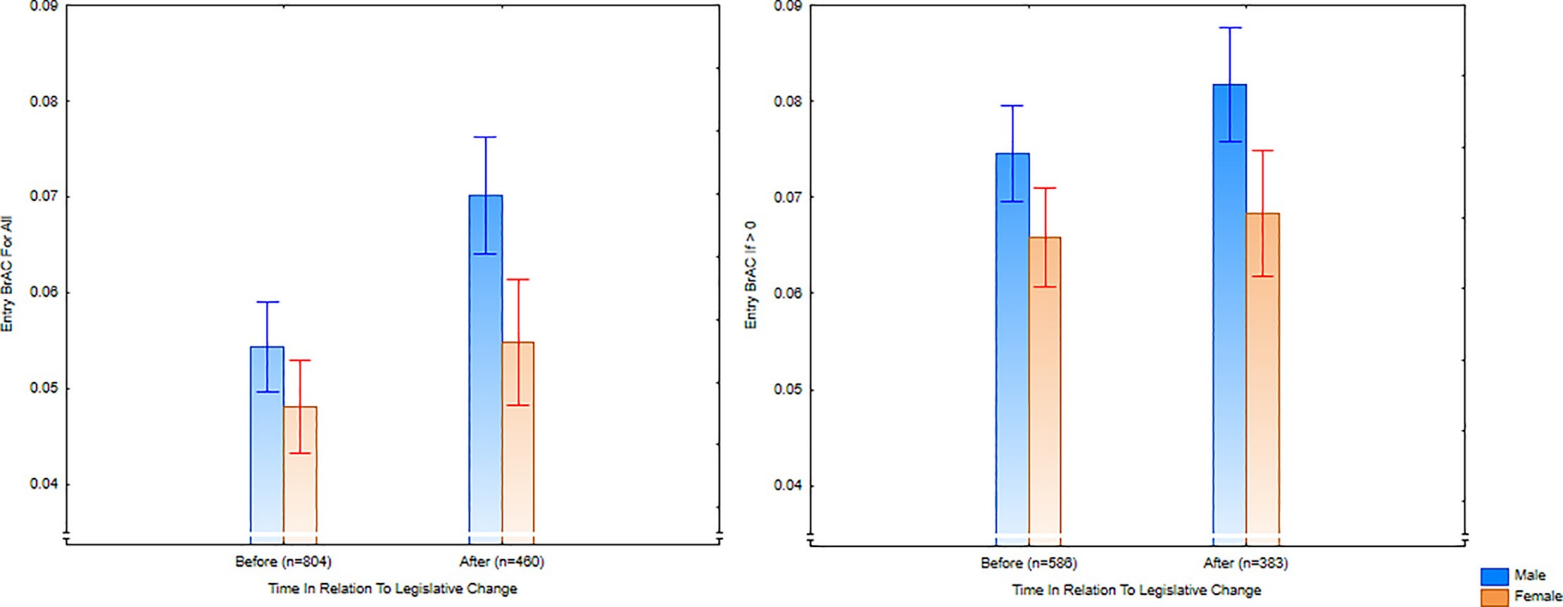
Entry breath alcohol concentration for January and February, before (2015–2016) and after (2017) the legislative changes, for all participants and only for drinkers.

Consistent with Study 2, while the proportion of people who entered the NED with a BrAC of zero remained the same over the baseline (2015 and 2016), it dropped significantly after the legislative change (*χ*^2^(*N* = 1,264, *df* = 1) = 17.6, two-tailed *p* < .0001, *Phi* = .12). Similarly, there was a stable baseline before the legislative change on entry BrAC, but an increase from before to after the legislation (Table 6; *F*(1,1262) = 16.8, *p* < .0001; *Hedges’ g* = 0.15, 95%CI: 0.04, 0.26). This was not just because of the greater numbers with zero BrAC before the legislative change: Those with a BrAC above zero at entry to NEDs tended to have lower levels before the new legislation compared to the sample after the legislative change, although this did not quite reach statistical significance (*F*(1,967) = 3.42, *p* = .065; See Fig 3). There was also an increase over time in participants admitting to taking ‘party drugs’ (*χ*^2^(*N* = 1,146, *df* = 1) = 16.01, two-tailed *p* < .0001, *Phi* = .12).

**Table 6:** January & February entry data for 2015, 2016 & 2017.

	2015			2016			2017		
Variable	Males (N = 347)	Females (N = 305)	All (N = 652)	Males (N = 67)	Females (N = 88)	All (N = 155)	Males (N = 246)	Females (N = 214)	All (N = 460)
Age in years $\bar{x}$ (sd; N)	22.93 (5.24; 345)	21.74 (4.44; 305)	22.38 (4.91; 650)	21.02 (3.44; 67)	20.26 (3.26; 88)	20.59 (3.35, 155)	21.71 (4.15; 246)	20.84 (4.66; 214)	21.31 (4.41; 460)
^a^Time of Night $\bar{x}$ (SD; Median; N)	22.37pm (.40; 22.37pm; 347)	22.39pm (.41; 22.35pm; 305)	22.38pm (.41; 22.36pm; 652)	22.57pm (.54; 22.47pm; 67)	22.34pm (1.00; 22.34pm; 88)	22.44pm (.59; 22.45pm; 155)	22.51pm (.55; 22.42; 246)	22.46pm (.50; 22.40pm; 214)	22.48 (.53; 22.42; 460)
Those admitting to having taken ‘party drugs’ before entry to NED. % (N)	3.5 (10)	1.88 (5)	2.72 (15)	7.14 (4)	8.98 (7)	8.21 (11)	11.38 (28)	7.48 (16)	9.57 (44)
Percentage of people with a BrAC of zero. % (N)	27.38 (95)	26.97 (82)	27.19% (177)	26.15 (17)	27.27 (24)	26.8 (41)	14.23 (35)	19.63 (42)	16.74 (77)
BrAC of People irrespective of alcohol use status. $\bar{x}$ (SD; Median, N)	.055 (.05; .048; 347)	.048 (.045; .042; 304)	.051 (.048; .044; 651)	.053 (.048; .047; 65)	.049 (.046; .039; 88)	.051 (.047; .043; 153)	.07 (.053; .067; 246)	.055 (.049; .047; 214)	.063 (.051; .055; 460)
BrAC Of Those With A BAC > 0% $\bar{x}$ (SD; Median, N)	.075 (.044; .072; 252)	.066 (.04; .059; 222)	.071 (.043; .066; 474)	.072 (.042; .065; 48)	.067 (.041; .056; 64)	.069 (.042; .061; 112)	.082 (.048; .08; 211)	.068 (.045; .059; 172)	.076 (.047; .07; 383)

Note

^a^ = Mean, median and standard deviation in hours and minutes.

Table 7 presents the data for people leaving the NEDs before and after legislative change. The samples came from people of an equivalent age range (*df* = 1010.94, *t*_upper_ -16.75<-1.65, *t*_lower_ 20.75>1.65) and the gender ratio was similar across years. The average time we assessed people leaving the NED was earlier after the legislative changes (*t*(1,014) = 16.72, *p* < .001). This contrasts with study 2, which found no difference over time.

**Table 7:** January & February exit data for2016 & 2017.

	2016			2017		
Variable	Males (N = 302)	Females (N = 200)	All (N = 502)	Males (N = 312)	Females (N = 202)	All (N = 514)
Age in years $\bar{x}$ (sd)	23.79 (5.29; 302)	22.48 (4.94; 200)	23.27 (5.18; 502)	22.9 (5.23; 312)	22.21 (4.67; 202)	22.63 (5.02; 514)
^a^Time of Night $\bar{x}$ (SD; Median)	2.42am (0.59; 2.44am)	2.20am (1.17; 2.16am)	2.33am (1.01; 2.34am)	1.47am (0.50; 1.48am)	1.53am (0.57; 2.02am)	1.49am (0.52; 1.53am)
Those admitting to having taken ‘party drugs’ while in the NED. % (N)	13.58 (41)	7.54 (15)	11.16 (56)	15.39 (48)	8.42 (17)	12.65 (65)
^b^Number Of Standard Drinks in town? $\bar{x}$ (SD; Median; N)	6.85 (4.79; 6.5; 26)	3.49 (3.1; 3; 39)	4.83 (4.17; 5; 65)	5.88 (4.77; 5; 312)	4.97 (4.26; 4; 202)	5.52 (4.59; 5, 514)
Percentage of people with a BrAC of zero. % (N)	6.95 (21)	12.5 (25)	9.16 (46)	12.18 (38)	14.85 (30)	13.23 (68)
BrAC of People irrespective of alcohol use status. $\bar{x}$ (SD; Median, N)	0.092 (.055; .094; 302)	.081 (.056; .082; 200)	.088 (.056; 502)	.083 (.06; .084; 312)	.086 (.06; .084; 202)	.084 (.058; .082; 514)
BrAC Of Those With A BAC > 0% $\bar{x}$ (SD; Median, N)	.099 (.051; .098; 281)	.093 (.051; .093; 175)	.097 (.051; .097; 456)	.095 (.051; .091; 274)	.101 (.053; .094; 172)	.097 (.052; .093; 446)

Note

^a^ = Mean, median and standard deviation in hours and minutes

^b^ = including those who reported to have had no drinks before inside the NED.

There was also a significantly higher ratio of people who had a BrAC of zero at the end of the night in 2017 compared with 2016, although this relationship accounted for only 0.42% of variance in the relationship (*χ*^2^(*N* = 1,016, *df* = 1) = 4.22, two-tailed *p* = .04, *Phi* = .06). The BrAC of patrons leaving the NEDs did not significantly differ across the years (*F*(1,1014) = 1.00, *p* = .32). Looking at the BrAC for patrons scoring above zero demonstrated equivalent results across the legislative change (*F*(1, 900) = 0.014, *p* = .91). Contrary to Study 2, reported drug use during the night also did not differ across the legislative changes (*χ*^2^(*N* = 1016, *df* = 1) = .54, *p* = .46, *Phi* = .02). In all, there was little change in exit data from before to after the legislation.

#### Assaults

Table 8 demonstrates that there was no significant difference in the pattern of crime across the dates of Study 3. While there was an 11% decrease in the absolute number of good order offences, there was a 6% increase in assaults. The same pattern of results was seen in Cairns (a large regional centre 1,700km north of Brisbane on the Great Barrier Reef, popular with young backpackers, which was subject to the State-wide 2 am closure outside NEDs). Cairns had a 29% decrease in good order offences (451 to 318), but a 12% increase in assaults over the same period (156 to 174). However, in the case of Cairns, this pattern of results was highly significant (*χ*^2^(*N* = 1,099, *df* = 1) = 12.08, *p <* .001, *Phi* = .11).

**Table 8:** Jan—Feb crimes against the person and good order offences.

		Good Order Offences	Assault
2016 (1^st^ January 2016 to 28^th^ February 2016) 2017 (1^st^ January 2017 to 28^th^ February 2017)	CBD—2016	347	65
	CBD—2017	352	86
	Valley—2016	437	67
	Valley—2017	343	54
	Total—2016	784	132
	Total—2017	695	140

No sig diff for totals: (*χ*^2^(*N* = 1,751, *df* = 1) = 1.85, *p* = .17, *Phi* = 0.03)

### Study 3 discussion

Assessing large numbers of people entering the NEDs over three years, Study 3 displayed consistent results with Study 2 in relation to preloading (Table 9). During the baseline from 2015 to 2016, there was no significant difference in the percentage of preloaders or the mean BrAC of people entering the NEDs, either as a whole, or only looking at those who had preloaded. However, there was a significant increase in the proportion of people who entered the NEDs with a BrAC above zero once the new legislation came into effect in 2017. Consistent with the idea that people were more likely to preload after the legislative changes (as predicted from Study 1), the time of night that people entered the NEDs became later over time.

**Table 9:** Summary table of results for studies 2 and 3.

Measure	Study 2 Result	Study 3 Result
Entry–Time of Assessment	Earlier**	Later**
Entry—Self-Reported Preloading	Increased*	—^a^
**Entry–Number with BrAC of Zero**	Decreased**	Decreased**
Entry—BrAC of those who are above zero	Increased**	No Change (p = .06)
**Entry—Mean BrAC of all participants**	Increased**	Increased**
Entry—Drug Preloading	Increased**	No Change (p = 0.28)
Exit–Time of Assessment	No Change (p = 0.63)	Earlier**
*Exit—number with BrAC of zero*	Decreased**	Increased*
**Exit—BrAC of those who are above zero**	**No Change**	**No Change**
Exit—Mean BrAC of all participants	Increased**	No Change (p = .26)
Exit—Drug Use in Town	Increased*	No Change (p = .46)
**Alcohol Related Crime**	**No Change**	**No Change**
Patron Presence (numbers of people)	Increase	—^a^

*p < .05

**p < .01

^a^ = untested; Bold = consistent results; Italics = inconsistent results.

Unlike study 2, the proportion of patrons with a non-zero BrAC at exit did not significantly increase after the legislation came into effect. Overall, there appeared to be very little change in BrAC at exit, which in combination with the entry data, suggests that patrons may be drinking in the NEDs to maintain a level of inebriation achieved through preloading. Also, and in contrast to Study 2, there was no change in the number admitting to taking drugs in Study 3.

The increased period of observation provided a better opportunity to test whether the legislation produced any difference in police records of alcohol-related violence on the publicly available database. While there was still no significant change in offences, the data suggested that a longer period of observation may have detected a reduction in good order offences but an increase in assaults. With a government-backed evaluation of the legislative changes currently underway [28], the researchers of the current, independent, evaluation were unable to obtain police data that would enable more fine-grained analysis of offences over time. However, we are also concerned that any changes in recorded offences may reflect changes in policing rather than being a direct outcome of the legislation. For example, the introduction of police body cameras coincided with the new legislation coming into effect and may have led to more warnings being issued before arrests were made. Any rise in arrests for assault may be due to reduced tolerance to public infractions. Alternatively, they may be due to the legislation resulting in large numbers of inebriated people being ejected from premises simultaneously at 3am.

## Overall discussion

In this research we aimed to build upon the work of Kypri et al (2010) [14] and measure violence, street populations and BrAC of people entering and leaving NEDs, before and after legislative change that restricted the availability of alcohol. We found that people predicted that the legislative change would either lead to them drinking more before and after entering the NEDs or that it would have no effect upon them. Based upon these results and theory [18] grounded in a motivation model of satisfying urges [17], we predicted that people would preload with more alcohol before entering the NED, leading them to come out even later and be more inebriated. We also predicted that this preloading would subvert the intention of the legislation, in that patrons would not show a reduction in average BrAC when they left licenced premises after the legislation came into force.

Results from the studies were consistent with our predictions, in that more people entered the NEDs having drunk alcohol, and the sample had a higher average BrAc following the introduction of legislation to reduce licencing hours for alcohol venues. Consistent with these increases in preloading, there was a noticeable reduction in the number of people entering the NED with a zero alcohol reading. Number of people who entered the NEDs with a BrAC reading of zero at least halved in both studies. The average BrAC of patrons attending the NED increased by 47.17% in Study 2 and 24% in Study 3. As this figure would be affected by the larger percentage of patrons preloading, we also looked only at those who had been drinking. Their average BrAC increased by 23.53% in Study 2, and 10.41% in Study 3. While the current research did not aim to experimentally test the scarcity heuristic, we argue that the phenomenon explains our results by increasing the intrinsic value of alcohol and leading to people preloading more before entry to the NED. As hypothesised, this also had the consequence of leading to a slightly later arrival time into the NED by patrons.

Consistent with a scarcity hypothesis, Study 2 found that patrons leaving the NEDs were significantly more inebriated after the legislative changes. To clarify the role of the scarcity heuristic or any other theory that explains the changes in behaviour, further study is required into people’s motivations for preloading and continuing to drink in the NEDs. Research into this is currently ongoing within our programme.

Study 3 found no difference over time in patrons’ inebriation at NED exit, even though on average they were leaving the NEDs earlier. Neither study saw a rise in the proportion leaving NEDs with a BrAC of zero after the legislation. In effect, it appears that patrons shifted their consumption towards preloading in both studies, while keeping their final BrAC either at the same as before the legislation or at an increased level. If the youth were motivated to titrate their drinking to achieve, and keep within, a certain upper inebriation level, then this would help to explain our results.

Regarding street counts, it appears that there were generally more people on the street in the 2017 arm of the Big Night Out than in the 2016 arm. This was also reflected in more people being inside Night Clubs in the 2017 arm of the study. It is difficult to know why this may be the case other than natural variation across different nights. With fear of assault not changing, we do not believe it was because people felt safer. One possibility is that there was a centralisation of patrons, with the more isolated NEDs (e.g., Caxton Street) becoming ‘dead-heart’ localities after midnight. Dead-heart cities lead to a centre that is unattended following a redistribution of the population, particularly after a working daytime crowd has left, or after a certain time frame. The legislative changes appeared to have had a larger effect on people who traditionally go out into the city in the early hours of the morning. The African community in Brisbane were one such subsample who used to attend the Caxton Street NED, and the main nightclub catering to them has subsequently closed their doors.

We did not have control sites in this study where the legislation did not apply, and so these results could, in principle, have been due to factors other than the legislative changes. That argument, however, inherently assumes that the legislation was ineffective to counteract other, unspecified, forces. However, it is difficult to conceive of an appropriate comparison site for this research. This was a State-wide legislative change (and, hence, other Queensland NEDs were subject to the same legislation) and other States (e.g., Victoria / NSW / Northern Territory) had their own specific cultures and legislative changes over the 3 years. A significant strength of the current research was that it was not undertaken or funded by government instrumentalities, the alcohol industry or registered health charities that promote reductions or abstention of alcohol consumption. This ensured that our research could be conducted with impartiality and integrity.

We argue that the current data provide a strong argument for their attribution to the legislative change. In particular, we had 3 years of data in Study 3 that showed a stable baseline in the important variables of preloading and entry BrAC. The one time there was a trend (i.e., entry time to the NED) we note this. The fact that the results of the current research coincided with the new legislation, after the stable baseline, would require that a substantial change in other determinants of alcohol consumption also occurred at that time, and we are aware of no evidence that this occurred. Since the legislation was introduced to reduce alcohol intoxication and assaults, the increase in preloading and the lack of changes in assaults within the areas studied in this research is troubling.

Furthermore, these results are consistent with recently published data [29] evaluating a crisis support service (the NightWatch) that operates in the same places as evaluated in the current studies. It was found in that research that there was the same amount of service utilisation by patrons visiting the NED, but that it had been moved to the beginning of the night, as opposed to spread more evenly throughout the night. It was also found that more intensive interventions (e.g., rest and recovery in a shelter) increased and interventions with less intensive requirements (e.g., Intoxication First Aid provided in situ on the street) were fewer. That is consistent with a growth in preloading and increased intoxication at the beginning of the night, while drinking across the night is curtailed.

We suggest that a simple reduction to alcohol availability through earlier closing times has had, to some degree, paradoxical effects on inebriation levels as people enter the city NEDs. As these effects are foreseeable, any legislation should encourage people to come out into the city NEDs earlier, reducing alcohol preloading and encouraging alcohol consumption in a controlled environment. Allowing the advertisement of ‘happy hour’ in places which also sell food and the use of entertainment enticements may be worth trialling. Once in the NED, we would suggest that to reduce alcohol consumption later in the night, it may be advisable to have alcohol pricing visually prominent in the pubs and clubs. Such an approach is consistent with Lynn (1989) [18]. We would also suggest that having breathalysing options within the NED may help to provide corrective feedback to people as to just how affected they had become.

Data on arrests during each evening hour were not available. However, it would be interesting to know whether assaults occurred around the 3am shut-out. If so, staged lockouts may be a better method of closing the NEDs. Either way, there appears to have been no reduction in peoples’ fear of assault while in the NED. Daily assault rates corroborate this result suggesting there has been little actual change. As noted, the legislative changes occurred at the same time that police were issued with body cameras, and we cannot be certain as to how these factors interacted.

In conclusion, we propose that paradoxical outcomes are best avoided by legislation that takes preloading and other cultural factors into account.

## Supporting information

S1 Data Data files in excel format. (ZIP)
